# Book Reviews

**Published:** 1800-03

**Authors:** 


### MEDICAL PHILOSOPHY.

*Physiologie philosophisch bearbeitet*, i. e. A Philosophic System of Physiology. By C. C. E. Schmid, &c.

[Continued from our last Rerrospect, pp. 181—184.]

In section IV. the learned author treats of the *Relation subsisting between Zoonomy and Zoology in general.* The latter is either considered in a historical view, when it relates to the description and history of animals; or in a philosophical view, when it deserves the name of Zoonomy. V. *On the form* (character) *of Zoonomia.* The peculiar form or character of zoological knowledge is the scientific, rational, and, properly speaking, philosophic; it comprehends the knowledge knowledge of all the phænomena occurring in animal life, digested according to the laws of the thinking faculty, the understanding and reason; it explains and reduces the illustrations to general principles and rules. General principles are the necessary conditions of rules and axioms, that is, of general conclusions. The essence of Zoonomy consists in representing and treating the phænomena in animals as the effect of one Nature, or as a total of the objects of perception systematically combined. For this purpose are required general representations of the uniform combination of what is manifold; or, in other words, of unity: there are natural rules necessary, if this variety is to be exhibited as a phænomenon to our sensible perception; and natural laws, if it claim at the same time the character of unexceptionable generality and necessity. The total, or complexus, of the phænomena, can only so far be conceived as one Nature, if we can comprehend them in systematic combination. The knowledge of the rules by which an object of sense is regulated, may be called physical knowledge; while the acquaintance with its laws, implies physical science. The principal objects of Zoonomy, considered as a physical science, are consequently, the rules and laws of animal nature. It is absolutely necessary, that the phænomena of animals, as well as all other objects, should constitute *one* nature, or rather that they be represented as connected by certain laws. For they are throughout objects of mental research; and our mind being the principle of all-comprehensive unity, it subjects every thing given within the sphere of its operation, every intuitive perception, to its combining activity, to rules and laws.

Section VI. *Farther elucidation of the idea relative to Zoonomia.* A self-preserving material part of nature is called an organic being. If such a being at the same time possess internal, that is, reflective activity, we call it an animal. An organic substance possessing merely external activity, is a plant. From this idea it follows, that, with a view of defining Zoonomia, and ascertaining its limits, there is required the knowledge of the following laws: 1. Of organic nature; 2. Of internally representing nature; and 3. Of their reciprocal relation to each other.

Section VII. *Preliminary knowledge of Zoonomia.* Under this head, the author ranks the historical knowledge of organic animal substance, in general.

Section VIII. *Constituent parts of Zoonomia.* In the natural constitution of an animal we can distinguish, 1. Organic nature in general; 2. The peculiar animal nature in particular.

Section IX. *Zoonomia considered as the science of vital power.* The power or activity of nature may be said to consist in that which we conceive as the foundation of the unity of determinate methods of combining diversified phænomena. That by which the organic nature of phænomena is determined, constitutes organic power; and that to which we ascribe the peculiarity of animal phænomena (life) may be called vital power. All Zoonomical knowledge ultimately is supported by something inconceivable; but this deficiency of explaining the original power, Zoonomy has in common with all physical science; and all human philosophy, and mental acquisition, in general, depend finally upon something, which, though it may be understood, cannot be comprehended and explained.

Section X. *Application of the idea combined with the term "science" to Zoonomia.* In a proper, strict sense of the word, Zoonomy is neither at present, nor can it ever become, a science. This idea is farther investigated by the author, and supported chiefly by the observation, that, according to the nature of the human faculties for acquiring knowledge, it is impossible to obtain any other but experimental or empirical information, from physical objects. In this section, as well as in the following, Prof. Schmid takes the opportunity of expressing his opinion on the Brunonian theory of medicine. *

Section XI. *Maxims by which a methodical Zoonomia ought to be regulated.* To reduce our present stock of Zoology to a scientific system, that is, Zoonomy, every thing depends upon the proper treatment of its objects; as the progressive improvements are boundless. The following are the rules which the methodical student of Zoonomy ought always to keep in view, and which appear to us equally just and acute, as they are obviously derived from mature sources:

1. All general ideas and laws must be abstracted from particular rules, and these again from individual observations and experiments.

2. Let the Zoonomist adopt only such a number of original animal powers, as is necessary to explain the phænomena resulting from them, as well as from the influence of other natural powers, with which we are already acquainted.

3. The Zoonomist should investigate only such substances as are conformable to experience, and can be exhibited as objects of sense, that is, simple bodies, mixtures, and compounds formed, to which he attributes the discovered powers.

4. A mere name, which denotes the unknown origin of a phænomenon, ought not to supply the place of the explanatory principle itself.

5. The Zoonomical inquirer, when he attempts to explain organic and animal phænomena, should renounce all useless and unfounded hypotheses, but particularly such as are of a metaphysical origin, and cannot be verified by experience.—This proposition is discussed in a manner peculiarly instructive.

6. The Zoonomist must endeavour to avoid all partial and incomplete explanations: consequently, he must expound no phænomenon of animal nature from a particular organ, without taking a view of the whole organized body; nor should he attempt the illustration of the entire organical structure, without attending to the influence of external agency. It is farther necessary, that, in the explanation of every phænomenon, the principles of physics, chemistry,

---

* An analysis of these profound observations would lead us too far from the present subject; but we propose to lay them before our readers in a future number of the next volume.

miſtry, mechanics, organization, and of life, ought to be ſyſtematically united, and their reſpective dependence on each other diſtinctly determined.—The excellent analyſis of this rule is accompanied with a remark on medical empiriciſm and rationaliſm. Mr. S. attempts to prove that, as we can learn the *materiale* of the phænomena in nature merely by the way of obſervation, and as we are unable to determine them a priori, *abſolute rationaliſm* does not, and cannot, exiſt in medicine. Nevertheleſs, the knowledge of animal nature, being the foundation of the medical art, is, according to his opinion, ſuſceptible of various degrees of philoſophical accuracy, conſequently of a continual approximation towards the idea of ſcientific execution — a ſyſtem. Hence the difference between an empirical and rational phyſician conſiſts only in degree, and can be aſcertained only by a due compariſon. The ſcale of the plus and minus ariſes from a more valuable ſtock of general ideas, the more extenſive circle of rules, the greater accuracy of explanation, and the ſyſtematic form of the whole.

XII. *Hiſtory of Zoonomy*; a very intereſting inquiry, which contains many inſtructive remarks; but our limited room does not permit us to give a tranſlation or an abridgment of this ſection.

[To be continued in our next Number.]

---

*Three Lectures upon Animal Life, delivered in the Univerſity of Pennſylvania. By* BENJAMIN RUSH, *M. D. &c.*

[Concluded from our laſt Number, pp. 184—187.]

After having treated of the different external ſtimuli, the learned author next proceeds to conſider the influence of internal ſtimuli on animal life. In this number he claſſes:

I. Food; which acts, 1. upon the tongue; 2. by maſtication; 3. by deglutition; 4, by its preſence in the ſtomach, both from its quantity and quality; and 5. by ſtimulating the whole body through the proceſs of digeſtion. II. The chyle. III. The blood. IV. A certain *tenſion* of the glands, and of other parts of the body. V. The exerciſes of the faculties of the mind. With reſpect to the laſt ſpecies of ſtimulus, the author obſerves that he ſees no occaſion to admit, with the followers of Dr. BROWN, that the mind is active in ſleep. He hopes to eſtabliſh the opinion of LOCKE, that the mind is always paſſive in ſound ſleep. "It is true," ſays he, "it acts in dreams, but theſe depend upon a morbid ſtate of the brain; and therefore do not belong to the preſent ſtage of our ſubject; for I am now conſidering animal life only in the healthy ſtate of the body. I ſhall ſay preſently, that dreams are intended to ſupply the abſence of ſome natural ſtimulus, and hence we find they occur in thoſe perſons moſt commonly in whom there is a want of healthy action in the ſyſtem induced by the exceſs or deficiency of cuſtomary ſtimuli."

In the ſecond Lecture, Dr. Ruſh inquires, with great ability, into the ſtate of animal life in ſleep—in the fœtus—in infants—in youth and middle life—in old age—in persons who are blind, deaf, and dumb—in idiots—in persons under the effects of long fasting—and in persons supposed to be dead from drowning, freezing, and other causes. When treating on the subject of sleep, the author relates the following anecdote:

"It is reported of Pope GANGANELLI, that he slept more soundly, and longer than usual, the night after he was raised to the papal chair. The effects of unusual sounds, in bringing on premature sleep, are farther demonstrated, by that constant inclination to retire to bed at an early hour, which country-people discover the first and second days they spend in a city, exposed from morning till night to the noise of hammers, files, and looms, or of drays, carts, waggons, and coaches, rattling over pavements of stone."

In the third Lecture, the author takes a view of the state of animal life, in the different inhabitants of the globe, as varied by civilization, diet, situation, and climate; he accounts for the influence of certain mental stimuli, which act nearly alike upon the individuals of all nations; of the causes of life in all the different classes of animals; of the causes of life in vegetables; of the causes of death; and lastly, he draws his conclusions from the doctrine of animal life, being the effect of impressions upon the body.

